# Long-Term Tolerability and Safety of AAV5*-Id3* Gene Therapy to Eyes

**DOI:** 10.1167/tvst.15.1.38

**Published:** 2026-01-28

**Authors:** Suneel Gupta, Rajnish Kumar, Nishant R. Sinha, Lynn M. Martin, Prashant R. Sinha, Frederick W. Fraunfelder, Alexandria C. Hofmann, Nathan P. Hesemann, Rajiv R. Mohan

**Affiliations:** 1 Harry S. Truman Memorial Veterans’ Hospital, Columbia, MO, USA; 2 Department of Veterinary Medicine & Surgery and Pathobiology and Integrative Biomedical Sciences, College of Veterinary Medicine, University of Missouri, Columbia, Missouri, USA; 3 Mason Eye Institute, School of Medicine, University of Missouri, Columbia, MO, USA

**Keywords:** AAV5, cornea, gene therapy, *inhibitor of differentiation 3* (*Id3*), and ocular toxicity

## Abstract

**Purpose:**

The *inhibitor of* *differentiation* *3* gene therapy via adeno-associated virus 5 (AAV5-*Id3*) effectively abrogated corneal fibrosis in vivo. This study examined the long-term tolerability and safety of AAV5*-Id3* gene therapy for eyes in vivo using a rabbit model*.*

**Methods:**

Eighteen New Zealand White rabbits, segregated into three groups (naive, AAV5-naked, and AAV5*-Id3*; *n* = 6/group), were used. The clinical eye examinations with slit-lamp and multimodal corneal imaging were performed in live rabbits periodically to record the status of ocular and corneal health for up to 7 months. Thereafter, humane euthanasia was performed, and cellular and molecular changes in corneas were studied employing histopathologic, immunofluorescence, and quantitative reverse transcription polymerase chain reaction (RT-PCR) techniques.

**Results:**

Periodic masked eye examinations with slit-lamp, Spectralis, HRT3-RCM, and specular ophthalmic microscopes found no significant differences in the corneas of naive, AAV5-naked, and AAV5*-Id3* groups. Modified McDonald–Shadduck scores revealed no signs of blepharospasm, chemosis, abnormal discharge, epiphora, erythema, or epithelial abrasion in three groups. Pachymetry, tonometry, and fluorescein testing detected no alterations in corneal thickness, intraocular pressure, and tear volume in eyes of the three groups. Histopathologic studies revealed corneal architecture, cellular organization, cellular morphology, and collagen levels similar in eyes of the three groups. Quantitative RT-PCR analysis did not find changes in nuclear factor κB, tumor necrosis factor α, α-smooth muscle actin, fibronectin, vascular endothelial growth factor, and pigment epithelium-derived factor messenger RNA levels in three test groups. At 7 months, AAV5-*Id3* corneas had 2.73 × 10^2^ ± 0.34 delivered-*Id3* gene copies*.*

**Conclusions:**

Targeted topical AAV5*-Id3* gene therapy is tolerable, safe, and nontoxic to the eyes in vivo.

**Translational Relevance:**

Topical AAV5*-Id3* gene therapy treats corneal fibrosis without significant side effects in vivo.

## Introduction

Blurred vision due to corneal fibrosis is common following ocular trauma or infection.^1^^–^^4^ Inflammation, excessive tearing, itching, neovascularization, and elevated intraocular pressure are additional morbidities associated with ocular injury.^5^^–^^10^ A plethora of research has shown the potential of many pharmacological and surgical methods to treat corneal fibrosis, as well as the underlying mechanism of action.^11^

Gene therapy is a promising modality to mitigate inherited and acquired corneal pathologies. The therapeutic genes can be topically administered into the cornea, and the impact of delivered genes in treated eyes can be visually monitored.^12^^–^^18^ Adeno-associated virus (AAV) vectors are the most common and used clinically in humans for ocular gene therapy applications.^12^^–^^21^ The single-stranded AAV vectors are safe for use in gene therapy since they cannot replicate on their own and need a helper virus to infect. Recombinant AAV used in gene therapy contains the DNA of the therapeutic gene, which is to be delivered. AAV vectors are found to deliver therapeutic genes effectively into corneal stromal keratocytes/fibroblasts in an effective and selective manner if introduced in the cornea via customized delivery techniques.^12^^–^^21^ Topical gene therapy in the cornea utilizing a suitable combination of vector and vector-delivery techniques has an acceptably low chance of systemic exposure.^12^^–^^18^ The potential of gene therapy for the treatment of many corneal problems, including herpetic stromal keratitis, hereditary dystrophies, allograft rejection, corneal fibrosis, and neovascularization, has been examined in the past decade.^5^^–^^9^^,^^22^^–^^27^ Most of these studies showed the efficacy of various genes in curing corneal pathologies in vivo in animal models but did not determine the long-term tolerability and safety of the tested genes.^12^^–^^16^ The testing of short- and long-term toxicity, tolerability, and safety is critical for the successful bench-to-bedside translation of corneal gene therapy approaches.

Accumulating literature shows the expression and role of *Id1*–*Id4* genes in human and animal corneas. The *Id* genes are shown to regulate cellular differentiation cascades by regulating transforming growth factor β (TGFβ)/Smad signaling through the basic helix–loop–helix (bHLH) protein and E-box sequences.^28^^,^^29^ TGFβ/Smad hyperactivity coordinates differentiation cascades via *Id3* and modulates the transcriptional mechanism underlying differentiation in corneal wound healing.^29^ Additionally, it is shown that localized, tissue-targeted AAV5-*Id3* gene therapy applied topically to the rabbit cornea effectively reduced corneal fibrosis without apparent acute toxicity.^13^ The present study evaluated the safety, toxicity, and tolerability of topical AAV5-*Id3* gene therapy in rabbit eyes in vivo for up to 7 months, employing periodic clinical eye examinations, multimodal corneal imaging in live animals, and histologic and molecular techniques following euthanasia.

## Materials and Methods

### Animals

The study was approved by the Institutional Animal Care and Use Committees (IACUC) of the University of Missouri and the Harry S. Truman Memorial Veterans’ Hospital and followed the ARVO Statement for the Use of Animals in Ophthalmic and Vision Research. Eighteen New Zealand White rabbits weighing 4 to 5 pounds acquired from the Charles River Laboratory (Wilmington, MA, USA) were used. Only one eye of each animal was used for the study, as per the IACUC guidelines. All ophthalmic procedures, clinical eye evaluations, and corneal imaging were performed under general anesthesia given via intramuscular injection of ketamine hydrochloride (50 mg/kg; JHP Pharmaceuticals, Rochester, MI, USA) and xylazine hydrochloride (10 mg/kg; XylaMed, Bimeda, Oakbrook Terrace, IL, USA). Before any procedure, two drops of a local anesthetic, ophthalmic proparacaine hydrochloride (0.5%; Alcon, Fort Worth, TX, USA), were applied onto the corneas. Sterile ophthalmic BSS drops (Alcon) were applied to the eyes during experiments to avoid corneal desiccation. At the endpoint, animals were humanely euthanized with an overdose of Euthaphen solution (150 mg/kg; pentobarbital sodium and phenytoin sodium; Dechra Veterinary Products, Overland Park, KS, USA) under general anesthesia.

### Chemicals and Surgical Supplies

High-quality research-grade chemicals/reagents were used in the study. The controlled substances and drugs were procured from the Veterans Affairs pharmacy (Harry S. Truman VA Medical Center, Columbia, MO, USA). Sterile Weck-Cel ophthalmic spears were obtained from Beaver-Visitec International (Waltham, MA, USA). Sterile surgical instruments, including forceps, wire speculum, and sharp Westcott scissors, were used from World Precision Instruments (Sarasota, FL, USA). Hematoxylin and eosin (H&E), Mason Trichrome, 2-methylbutane, and other chemicals were acquired from StatLab Medical Products (McKinney, TX, USA) or Thermo Fisher (Waltham, MA, USA). Antifade mounting medium with DAPI was procured from Vector Laboratories (Burlingame, CA, USA).

### AAV5-*Id3* Development

*The Id3* gene was cloned into the AAV5 plasmid pTRUF11 after quantitative real-time polymerase chain reaction (qRT-PCR) amplification from rabbit corneal fibroblast complementary DNA (cDNA) as described earlier.^13^ In brief, the cytomegalovirus enhancer, chicken-actin promoter (hybrid promoter), and simian virus 40 polyadenylation site were all included in the viral expression cassette. A two-plasmid cotransfection packaging technique, as previously reported, was used to create the recombinant AAV5*-Id3* virus.^13^

### AAV5-*Id3* transduction

AAV5*-Id3* transduction was administered to the corneal stroma as reported previously.^12^^–^^18^ The animals were divided into three groups, each containing six animals. Group 1 (*n* = 6; naive group) corneas were naive controls, group 2 (*n* = 6; naked-vector) received 100 µL AAV5-naked vector titer (3.8 × 10^12^ µg/mL), and group 3 (*n* = 6; AAV5*-Id3*) received 100 µL AAV5*-Id3* titer (6.5 × 10^12^ µg/mL) for 5 minutes via a customized cloning cylinder technique. The corneal epithelium was gently scraped off with a No. 64 Beaver blade (BD Biosciences, Franklin Lakes, NJ, USA), the deepithelialized cornea was dried with Weck-Cel, and thereafter viral titer was administered. The procedures were performed under a surgical microscope.

### Clinical Eye Examinations and Multimodal Corneal Imaging

Anesthetized rabbits underwent clinical eye examinations and corneal imaging before and after AAV vector administration after 30 minutes, 3 days, 7 days, 14 days, 21 days, 1 month, 2 months, 3 months, 5 months, and 7 months (and in between if needed). At least three observers (SG, NRS, LMM, NPH, ACH, or RRM) independently assessed eye health in a masked manner.

The clinical eye health evaluation data were recorded using a slit-lamp microscope (SL-15 portable slit lamp; Kowa, Torrance, CA, USA) equipped with a high-definition digital imaging system (Kowa, portable VK-2 Ver. 5.50), as described previously.^12^^–^^18^ Moreover, corneas were evaluated with a stereomicroscope (Leica MZ16F; Leica Microsystems, Buffalo Grove, IL, USA) fitted with a digital camera (SpotCam RT KE; Diagnostic Instruments, Sterling Heights, MI, USA) as described previously.^12^^–^^18^ In addition, the subjective evaluation of the rabbit eyes was performed with an indirect ophthalmoscope (Keeler All Pupil II Wired Indirect; Keeler Ltd., Berkshire, UK) as described.^30^

The multimodal two-dimensional/three-dimensional corneal imaging was performed with the high-resolution confocal imaging systems SPECTRALIS (Heidelberg Engineering, GmBH, Dossenheim, Germany) and HRT3-RCM anterior segment module (Heidelberg Engineering, GmBH) to compare corneal anatomy, cellular density, conjunctiva, and limbus structures as described previously.^17^^,^^18^ Corneal endothelial cell morphology and density were assessed with a noncontact specular microscope (NSP-9900; Konan Medical, Irvine, CA, USA). The captured images were used for endothelial cell density determination and morphometric analysis using the fixed-frame method, quantitative analysis of cell structure, coefficient of variation, and percentage of hexagonal cells.^17^^,^^18^

### Corneal Epithelial Health Assessment

An ophthalmic fluorescein dye (Altafluor Benox, an orange-red diagnostic solution) was applied topically to evaluate the changes in the corneal epithelial layer, record corneal abrasions, and detect foreign bodies with a stereomicroscope (Leica MZ16F) equipped with a digital camera system (SpotCamRT KE; Diagnostic Instruments). The observations were made using a GFP light filter as described previously.^12^^–^^18^

A modified McDonald–Shadduck ocular grading system gauged the toxicity of applied AAV5-naked and AAV5-*Id3* vectors.^16^^,^^18^^,^^31^ Tonometry measured the intraocular pressure (IOP) in rabbits via a tonometer (Tono-Pen AVIA; Reichert Technologies, Depew, NY, USA) while central corneal thickness (CCT) was assessed by pachymeter (Accu Pach VI Pachymeter; Accutome, Malvern, PA, USA) and tear volume by the Schirmer test strips (Thermo Fisher) as reported earlier.^30^ To minimize variability, a single observer (SG) conducted all IOP data between 9 AM and 12 PM, accounting for both operator consistency and diurnal fluctuations.^13^^–^^18^ An average of 10 readings was recorded for the analysis.

### Corneal Tissue Collection

The corneal tissues were collected 7 months post-AAV topical application using surgical forceps and Westcott scissors. The corneas were either snap-frozen in liquid nitrogen or placed into cryomolds containing an optimal cutting temperature compound and stored at –80°C. Tissues were cut into 8-µm-thick sections using a cryo-microtome, mounted on glass microscopic slides (Superfrost Plus; Thermo Fisher), and stored at –80°C for histology.^12^^–^^18^

### Messenger RNA Extraction, cDNA Synthesis, and qRT-PCR

The messenger RNA (mRNA) was extracted from naive and 7-month post-AAV5-treated rabbit corneas following vendor instructions using a commercial kit (Promega, Madison, WI, USA) and reverse transcribed into cDNA using an RNeasy kit (Qiagen, Valencia, CA, USA).^12^^–^^18^ The real-time qRT-PCR reactions were performed using a SYBR green supermix (Bio-Rad Laboratories, Hercules, CA, USA) in the QuantStudio 6 Flex Real-Time PCR System (Thermo Fisher). Each 20-µL reaction contained 10 µL 2× SYBR green supermix, 2 µL cDNA (0.5 µg), 2 µL forward primer (0.2 µM), 2 µL reverse primer (0.2 µM), and 4 µL DNase/RNase free water and ran at a universal cycle (95°C for 3 minutes and 40 cycles of 95°C for 30 seconds, followed by 60°C for 60 seconds) following a published protocol.^12^^–^^18^^,^^31^^–^^35^ The mRNA expressions of selected inflammatory genes nuclear factor κB (NF-κB) and tumor necrosis factor α (TNF-α), fibrotic genes α-smooth muscle actin (α-SMA) and fibronectin (FN), and angiogenic regulators vascular endothelial growth factor (VEGF) and pigment epithelium-derived factor (PEDF) were evaluated. The primer nucleotide sequences (forward and reverse) used in the study are listed in Table 1. Beta-actin (β-actin) was used as a housekeeping gene for normalization of data.

**Table 1.** List of the Primers Used for mRNA Expression Analysis and Gene Copy Number Analysis

Gene	Gene Abbreviation	5′-3′ Forward Primer	5′-3′ Reverse Primer	Accession Number
For mRNA expression analysis				
Beta-actin	β-actin	CGGCTACAGCTTCACCACCA	CAGGCAGCTCGTAGCTCTTC	X_00351
Nuclear factor kappa B	NF-κB	AGTGCTGGAGTTCAGGATAAC	GAGAATGAAGGTGGATGATTGC	NM_001261403.3
Tumor necrosis factor alpha	TNF-α	CCCAGGCAGTCAGATCATCTTC	AGCTGCCCCTCAGCTTGA	U42625.1
α-Smooth muscle actin	α-SMA	GGGTGACGAAGCACAGAGC	CTTCAGGGGCAACACGAAGC	NM_001613
Fibronectin	FN	CGCAGCTTCGAGATCAGTGC	TCGACGGGATCACACTTCCA	NM_002026
Vascular endothelial growth factor	VEGF	ACCCATGGCAGAAGAAGGAGACAA	ACTCCAGGCTTTCATCATTGCAGC	AF024710.1
Pigment epithelium-derived factor	PEDF	TGATGTCGGACCCTAAGGCTGTTT	ATGAATGAACTCGGAGGTGAGGCT	NM_002615.4
For gene copy number analysis				
Inhibitor of differentiation	AAV5-Id3	CGCGTCATCGACTACATTCTC	CCCATGGTCTTCTTCTGCATT	Custom Oligos

### Measurement of Gene Copies

To determine copies of the delivered *Id3* gene in the rabbit cornea, PCR reactions were performed using the reported method.^16^ The corneas were ground in liquid nitrogen, and genomic DNA was isolated (Qiagen DNeasy kit). The standard curves were created by 10-fold serial dilution of plasmid with the *Id3* gene (10^0^–10^8^/µg DNA). Then, 20-µL PCR reactions were run at 95°C for 10 minutes, 40 cycles at 95°C for 15 seconds, and 60°C for 1 minute using the forward and reverse primers provided in Table 1.

## Histology and Fluorescence Microscopy

### H&E Staining, Masson's Trichome, and Keratocan Staining

H&E and Masson's trichrome staining were applied to corneal sections in our laboratory using a published method.^12^^–^^18^

Keratocan (KERA) staining was performed using the goat KERA antibody raised against the peptide H2N-LRLDGNEIKPPIPIDLVAC-OH (0.6 mg/mL; a gift from Prof. Winston Kao) as the primary antibody, and immunostaining was performed on rabbit corneal sections. In brief, for KERA staining, corneal tissue sections were incubated at room temperature with the primary antibody at a 1:100 dilution in a 1 HEPES buffer containing 2% bovine serum albumin for 90 minutes, followed by donkey anti-goat IgG secondary antibody (Alexa Fluor 594; A11058; Invitrogen, Carlsbad, CA, USA) at a 1:500 dilution for 60 minutes. After completion of immunostaining, tissue sections were mounted in a medium containing DAPI (Vectashield, Newark, CA, USA), viewed, and photographed under a fluorescence microscope (Leica, Deerfield, IL, USA) equipped with a digital camera system (SpotCam RT KE, Sterling Heights, MI, USA).^36^^–^^40^

A TdT-dUTP terminal nick-end labeling assay was performed according to the manufacturer's instructions and as reported previously^12^^–^^18^ to detect the apoptotic cells. In brief, the tissue sections were fixed in 1% paraformaldehyde at room temperature, followed by subsequent permeabilization with ethanol/acetic acid (2:1 ratio; at 20°C) and treated with fluorescent detection assay kit reagents to detect apoptosis and/or necrosis.

### Statistical Analysis

Statistical analysis involved one-way analysis of variance followed by a Bonferroni or Tukey post hoc multiple comparison test. The standard error of the mean was used to express the average values. *P* ≤ 0.05 was considered statistically significant.

## Results

### In Situ Clinical Biomicroscopy Ocular Examinations in Live Animals

**Figure 1.**
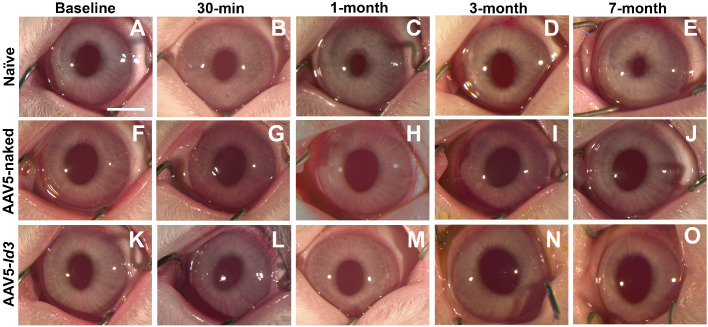
Representative stereobiomicroscopic images showing corneal health in naive, AAV5-naked, and AAV5-*Id3* groups at baseline, 30 minutes, 1 month, 3 months, and 7 months posttreatment. Corneal appearance remained comparable across the naive (**A–E**), AAV5-naked (**F–J**), and AAV5-*Id3* (**K–O**) groups throughout the study. AAV5-*Id3* gene therapy induced no observable long-term corneal toxicity (*P* > 0.05). *Scale bar*: 2.0 mm.

**Figure 2.**
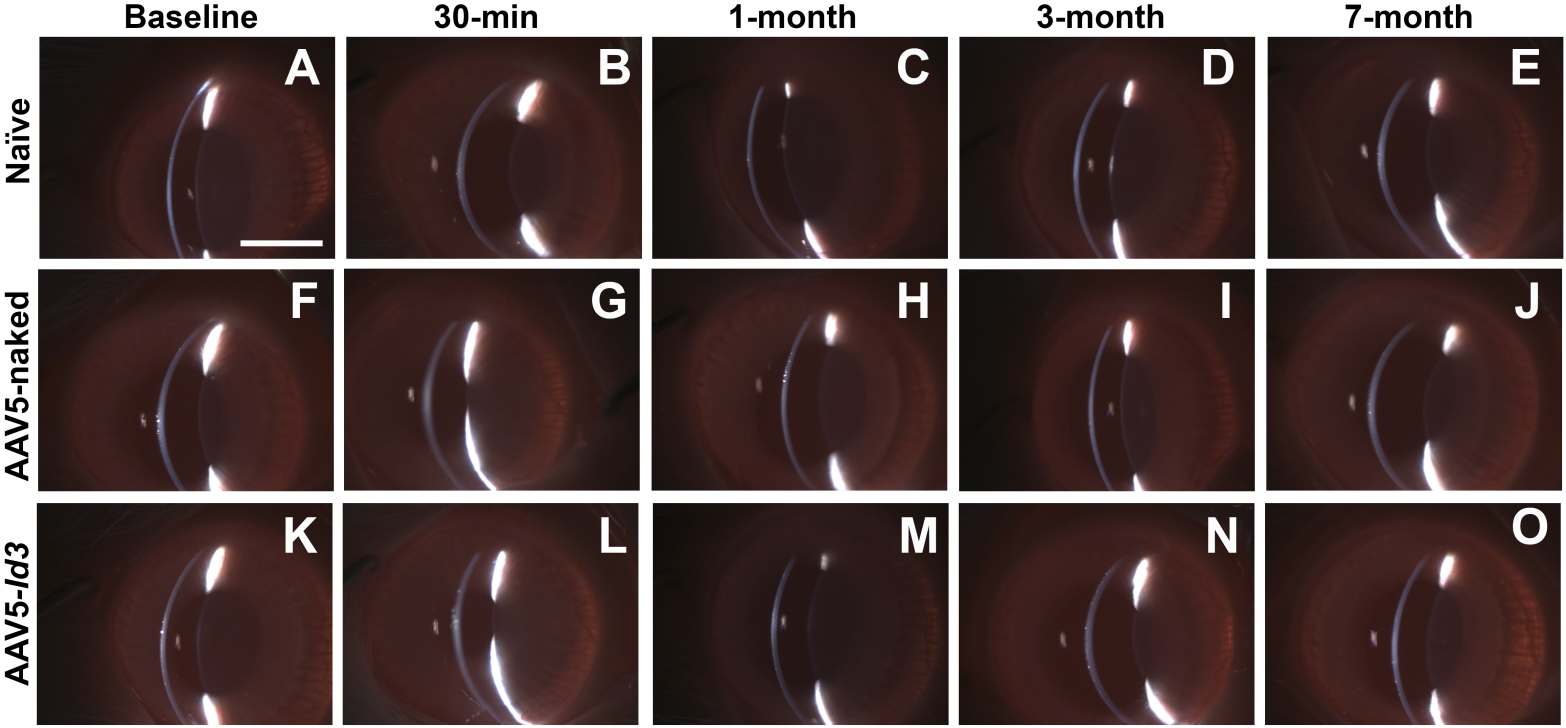
Representative slit-lamp narrow-beam images demonstrating the long-term safety and tolerability of AAV5-*Id3* gene therapy in rabbit eyes over 7 months. Slit-beam analysis revealed no evidence of corneal opacity or inflammation in the AAV5-*Id3* group (**K–O**), with corneal appearance comparable to AAV5-naked (**F–J**) and naive (**A–E**) controls. *Scale bar*: 2.0 mm.

**Figure 3.**
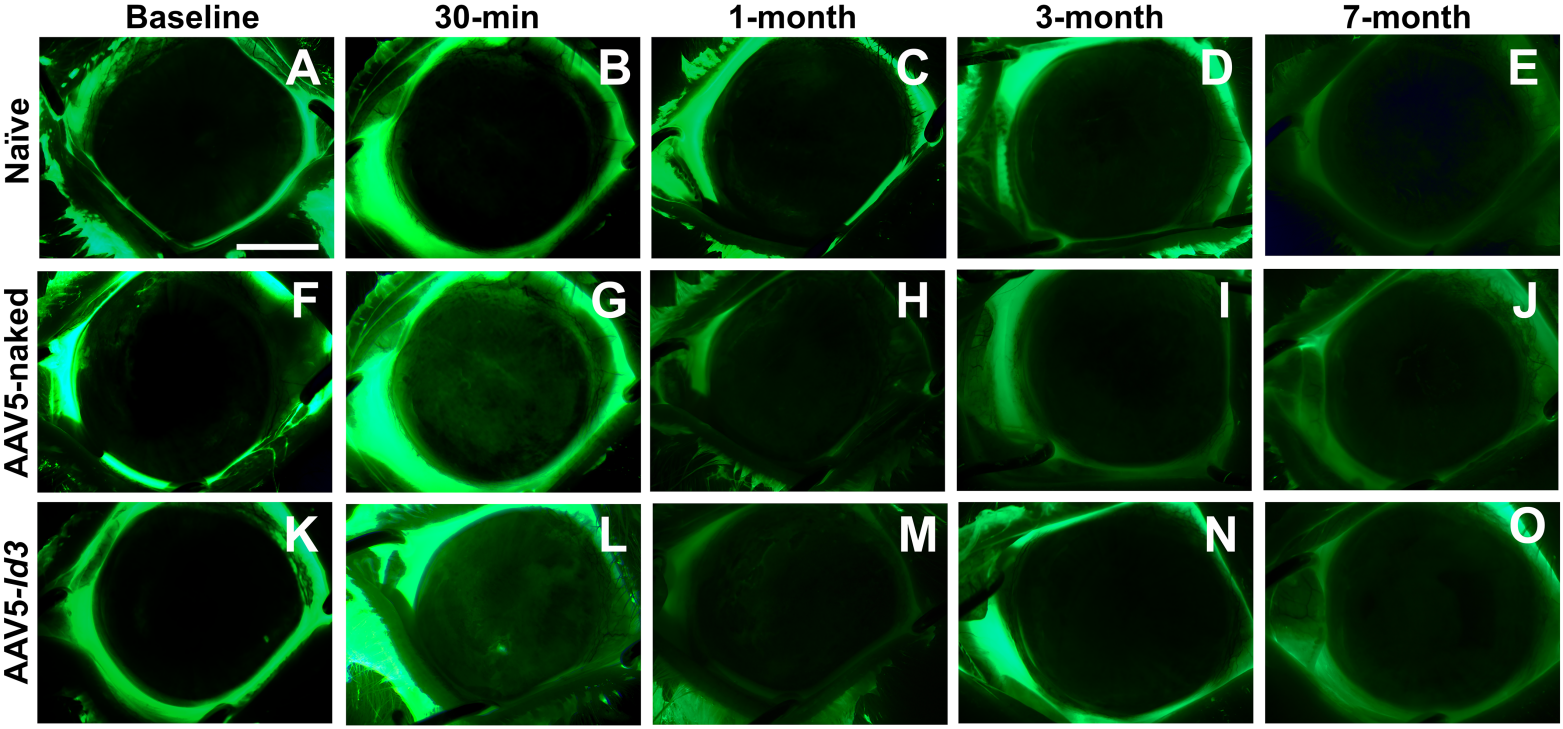
Representative fluorescein staining images showing corneal epithelial health in rabbit eyes following *AAV5-Id3* gene therapy over 7 months. No evidence of corneal abrasion or epithelial defects was observed in the AAV5-*Id3* group (**K–O**), with staining patterns comparable to naive (**A–E**) and AAV5-naked (**F–J**) controls. *Scale bar*: 2.0 mm.

Clinical eye examinations of the naive, AAV5-naked, and AAV5-*Id3* rabbits were performed over 7 months posttreatment. No eyes in any three groups showed clinically significant ocular inflammation, corneal defects (haze, edema, or neovascularization), redness, laceration, or chemosis during ophthalmic examinations (Figs. 1, 2). Also, the sclera, conjunctiva, and eyelid showed no any clinical abnormalities (Fig. 2). The fluorescein dye test did not detect any defect in corneal epithelial of the three groups over the longest tested 7-month time point (Fig. 3). In addition, an indirect ophthalmoscope subjective evaluation of the posterior chamber of the rabbits eye did not show any noticeable changes in between the groups as compared with naive controls.

### In Situ Clinical Confocal Microscopy Examinations in Live Animals

**Figure 4.**
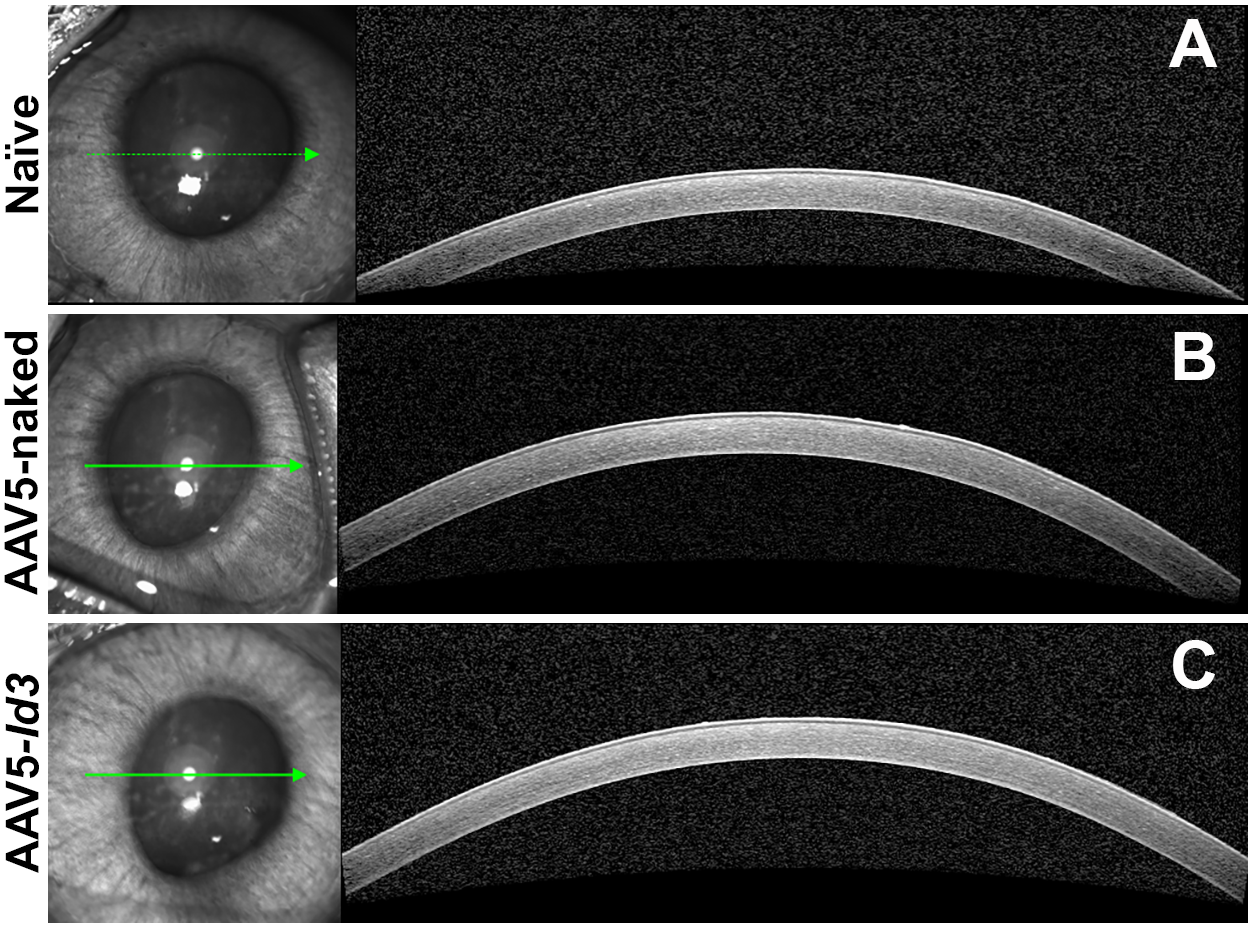
Representative Spectralis anterior segment module (Spectralis-ASM) confocal cross-sectional images showing corneal tissue morphology across naive (**A**), AAV5-naked (**B**), and AAV5-*Id3* (**C**) groups. No significant differences in corneal thickness or structural anomalies were observed between groups (*n* = 6 per group; *P* > 0.05).

**Figure 5.**
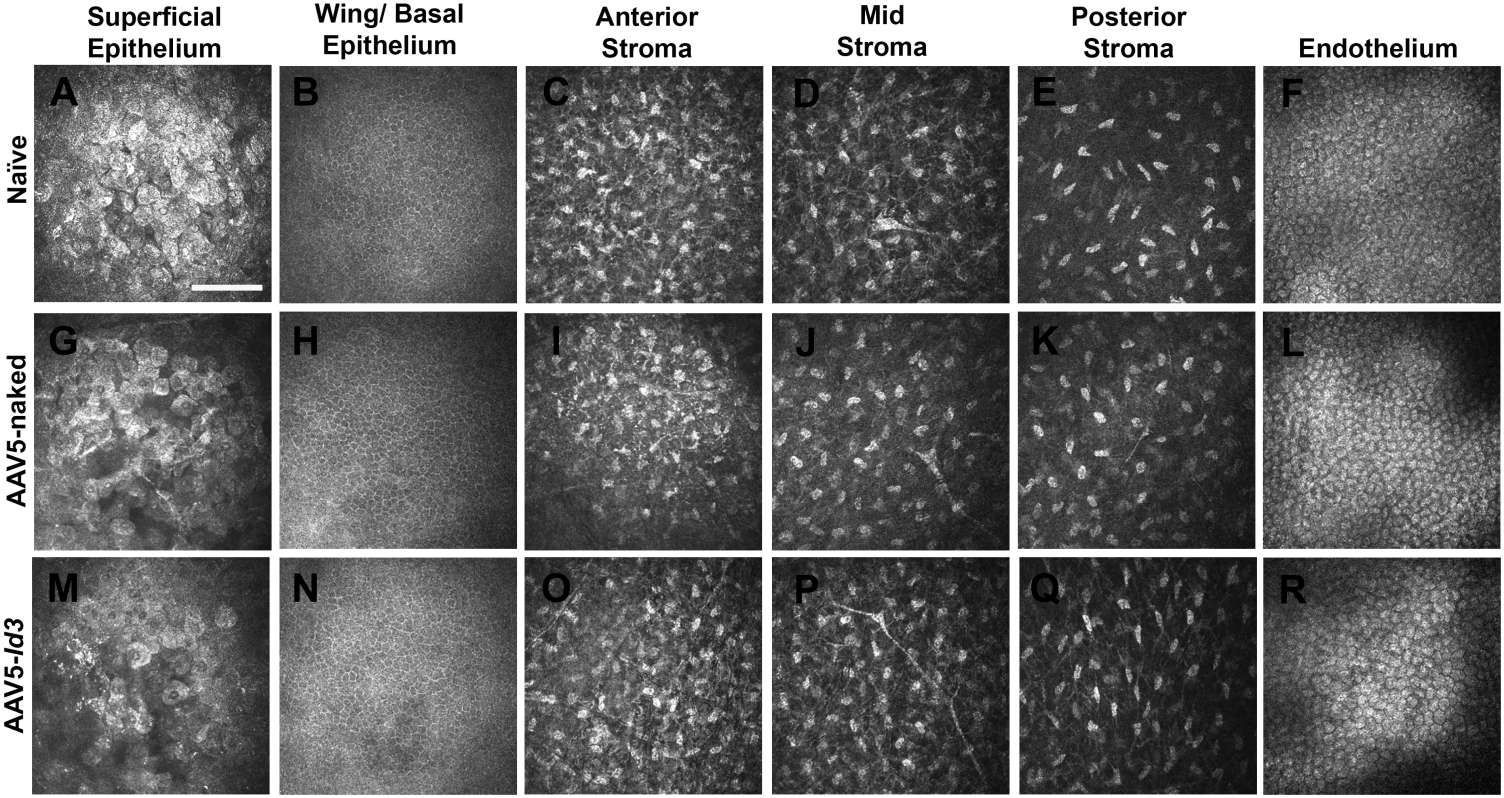
Representative in vivo confocal microscopy images of the superficial epithelium, wing/basal epithelium, anterior stroma, mid-stroma, posterior stroma, and endothelium of rabbit corneas from naive (**A–F**), AAV5-naked (**G–L**), and AAV5-*Id3* (**M–R**) groups at 7 months posttreatment. Images from the AAV5-*Id3* group showed no appreciable differences compared to AAV5-naked and naive controls (*n* = 6 per group; *P* > 0.05). Each image represents a 400-µm × 400-µm area. *Scale bar*: 100 µm.

**Figure 6.**
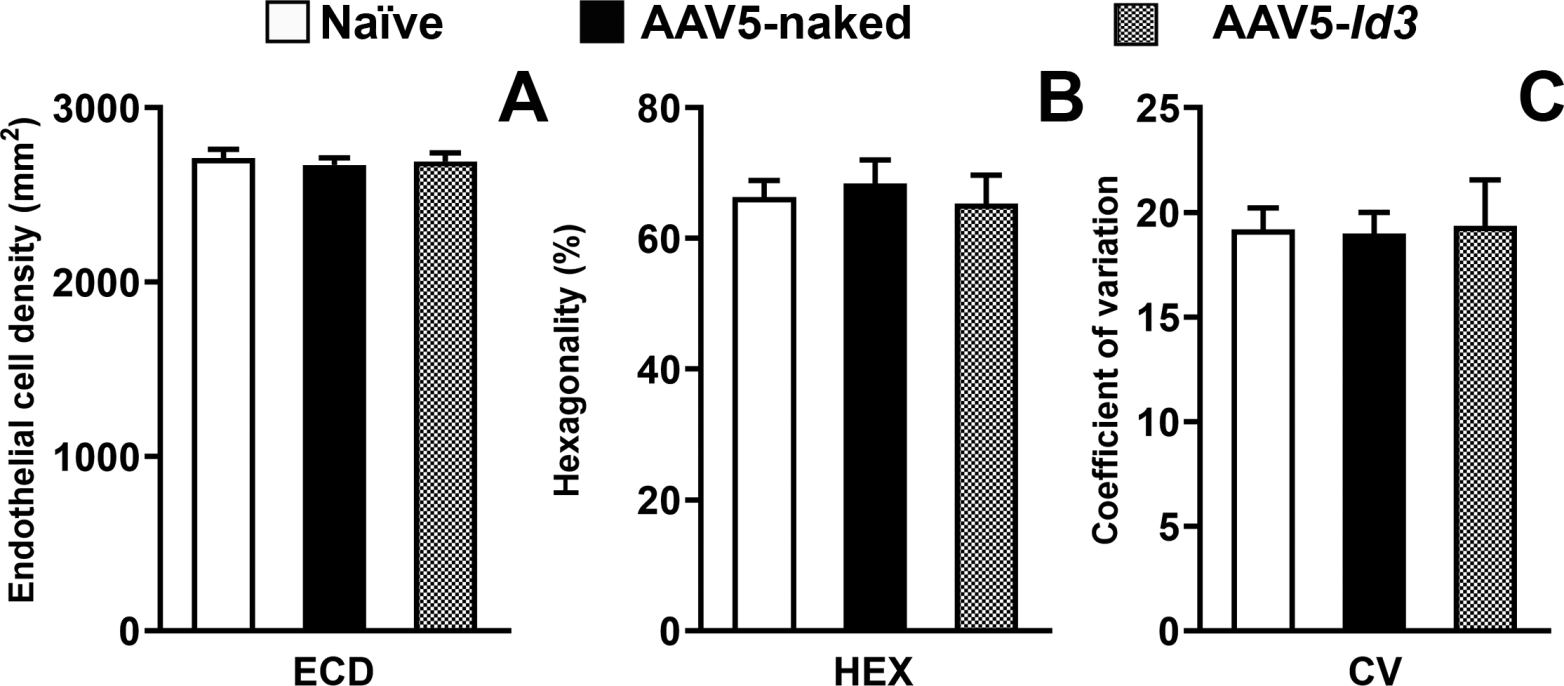
Bar graphs showing corneal endothelial cell density (ECD, **A**), hexagonality (HEX, **B**), and coefficient of variation (CV, **C**) in naive, AAV5-naked, and AAV5-*Id3* rabbit eyes. No significant differences were observed among groups, demonstrating the safety and tolerability of AAV5-*Id3* gene therapy in vivo. Statistical comparisons were performed using one-way analysis of variance with Tukey's post hoc test (*P* > 0.05).

A noncontact, detailed visualization of the corneal tissue was performed using confocal optical coherence tomography with the Spectralis microscope (Franklin, MA, USA). The cross-sectional high-resolution axial images of the naive, AAV5-naked, and AAV5-*Id3* groups showed a similar corneal tissue morphology without clinical irregularities (Fig. 4). Similarly, the HRT3-RCM microscopic examinations of corneas found no significant defects in corneal epithelium, stroma, endothelium, or corneal architecture for 7 months (Fig. 5). The confocal images of the superficial and wing/basal corneal epithelium of the naive (Figs. 5A, 5B) and AAV5-naked (Figs. 5G, 5H) rabbits resembled the AAV5-*Id3* (Figs. 5M, 5N) rabbit eyes at 7 months in vivo. In all three groups, corneal epithelial cells had normal morphology. The in vivo images for the stroma (anterior, mid, and posterior) of AAV5-*Id3* group rabbit corneas (Figs. 5O, 5Q) appeared similar to those of AAV5-naked (Figs. 5I, 5K) and naive (Figs. 5C, 5E) corneas. The corneas of naive, AAV5-naked, and AAV5-*Id3* had normal keratocyte morphology and cell density in the anterior stroma up to 7 months the longest tested time points. Likewise, posterior stroma had keratocyte morphology and cellular density similar in AAV5-*Id3* (Fig. 5Q), AAV5-naked (Fig. 5K), and naive (Fig. 5E) corneas. All rabbit naive, AAV5-naked, and AAV5-*Id3* corneas displayed a characteristic single layer of hexagonal endothelial cells with comparable size and shape in naive (Fig. 5F), AAV5-naked (Fig. 5L), and AAV5-*Id3* (Fig. 5R) corneas at 7 months. No signs of pleomorphism or polymegethism were observed in naive, AAV5-naked, and AAV5-*Id3* corneas. The specular microscopy found no significant changes in cell density (*P* = 0.469; Fig. 6A), hexagonality (*P* = 0.375; Fig. 6B), and coefficient of variation (*P* = 0.439; Fig. 6C) of the corneal endothelium of the naive, AAV5-naked, and AAV5-*Id3* rabbit corneas.

### Modified McDonald–Shadduck Evaluations

A modified McDonald–Shadduck test found AAV5-*Id3* gene therapy tolerable, nontoxic, and safe to the eyes. No noticeable morbidities to the cornea, eyelid, conjunctiva, and iris were observed over 7 months based on scores for pupillary light reflex, conjunctival issues (swelling, congestion, and discharge), and corneal issues (percentage opacity, corneal haze, and neovascularization) (Table 2). The cumulative scores did not reveal any significant abnormal changes among the three groups (*P* > 0.05).

**Table 2.** Modified McDonald–Shadduck Scores Showing the Comprehensive Ocular Health Assessment

	Modified MacDonald–Shadduck Score			
Groups	Baseline	1 Month	3 Months	7 Months
Naive	0	0	0	0
AAV5-naked	0	0.87 ± 0.08	0.75 ± 0.05	0.67 ± 0.06
*AAV5-Id3*	0	1.01 ± 0.11	0.81 ± 0.06	0.91 ± 0.07

Cumulative scores are shown as the mean ± standard error of the mean.

### CCT, IOP, and Tear Evaluations

**Figure 7.**
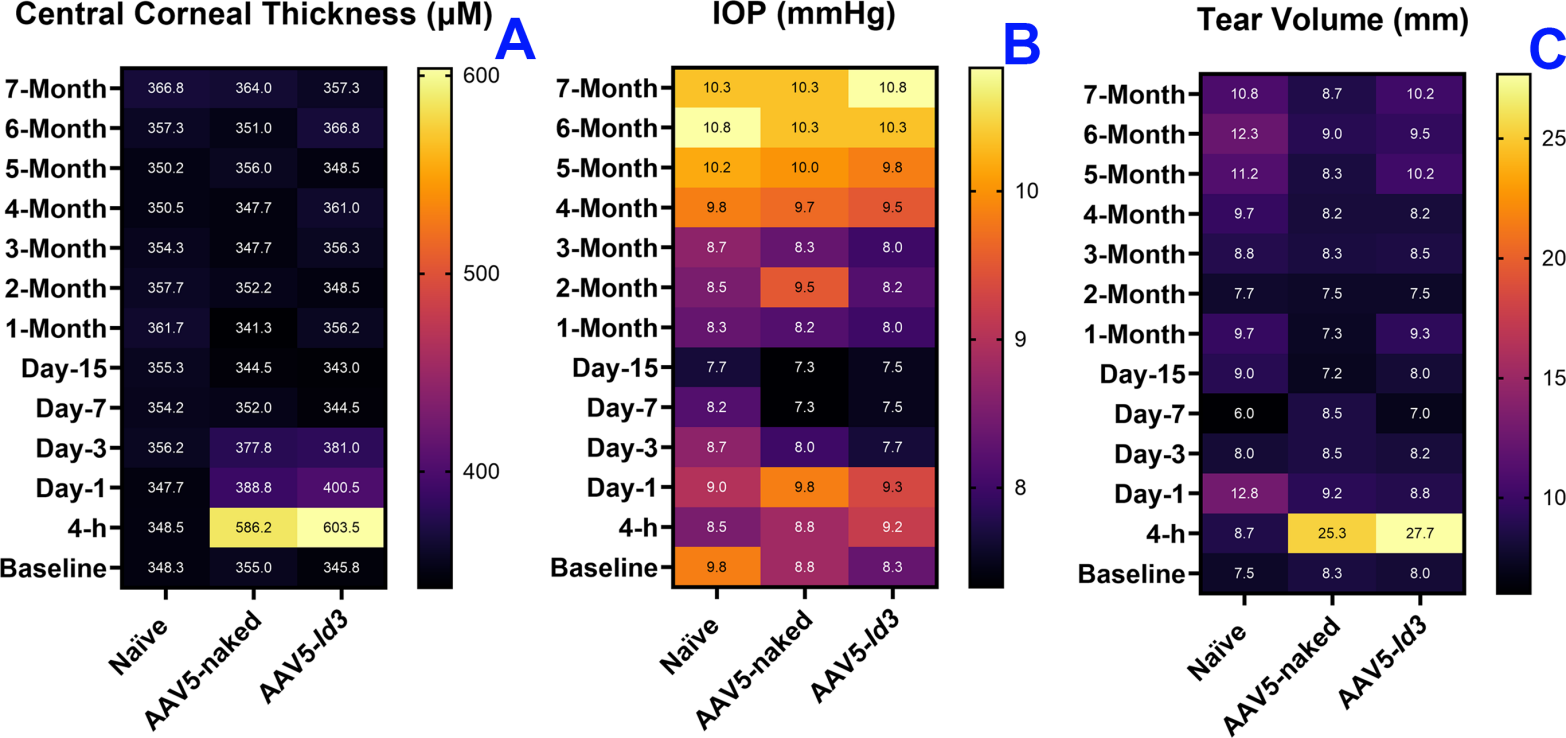
Heatmaps showing CCT (**A**), IOP (**B**), and tear volume (**C**) over time in naive, AAV5-naked, and AAV5-*Id3* rabbit eyes. AAV5-*Id3* gene therapy had no significant impact on CCT, IOP, or tear production compared to controls, demonstrating long-term ocular safety. Statistical comparisons were performed using two-way analysis of variance with Bonferroni post hoc test (*P* > 0.05).

The mean CCT in naive, AAV5-naked, and AAV5-*Id3* eyes was 348.3 ± 8.29 µm, 355.0 ± 7.31 µm, and 345.8 ± 9.32 µm, respectively, before the procedure. The CCT values for the naive, AAV5-naked, and AAV5-*Id3* eyes after 7 months were 357.3 ± 8.19 µm, 364.0 ± 7.19 µm, and 366.8 ± 9.48 µm, respectively. As expected, CCT increased temporarily in response to epithelium removal in the treatment groups and returned to baseline as reepithelialization occurred over the following 3 days. The AAV5-*Id3* or AAV5-naked vector did not significantly alter CCT throughout the course of the investigation (Fig. 7A).

The mean IOP in naive, AAV5-naked, and AAV5-*Id3* rabbit eyes ranged from 9.76 ± 0.69 to 10.1 ± 1.21 mm Hg prior to the procedure. During the entire course of 7 months, the IOP values ranged between 7.3 ± 1.11 and 10.8 ± 1.03 mm Hg in all groups (Fig. 7B). This indicated that AAV5-*Id3* therapy in the cornea did not pose a risk for glaucoma.

The mean tear flow in the naive, AAV5-naked, and AAV5-*Id3* rabbit eyes was 7.5 ± 3.11 mm, 8.3 ± 2.17 mm, and 8.0 ± 1.27 mm, respectively, prior to gene delivery (Fig. 7C). An expected momentary increase in tears was detected after gene delivery (AAV5-naked had 25.3 ± 4.21 mm and AAV5-*Id3* had 27.7 ± 3.87 mm), but it reverted to normal after 3 days. This leap was due to mild damage to the corneal epithelium because of the vector-delivery technique. Within 3 days, tear values in AAV5-*Id3* and AAV5-naked eyes returned to normal (8.5 ± 2.01 and 8.2 ± 1.87, respectively). Thereafter, no significant differences in tears were detected among naive, AAV5-naked, and AAV5-*Id3* eyes over 7 months.

### Histologic Corneal Evaluations Following Euthanasia

**Figure 8.**
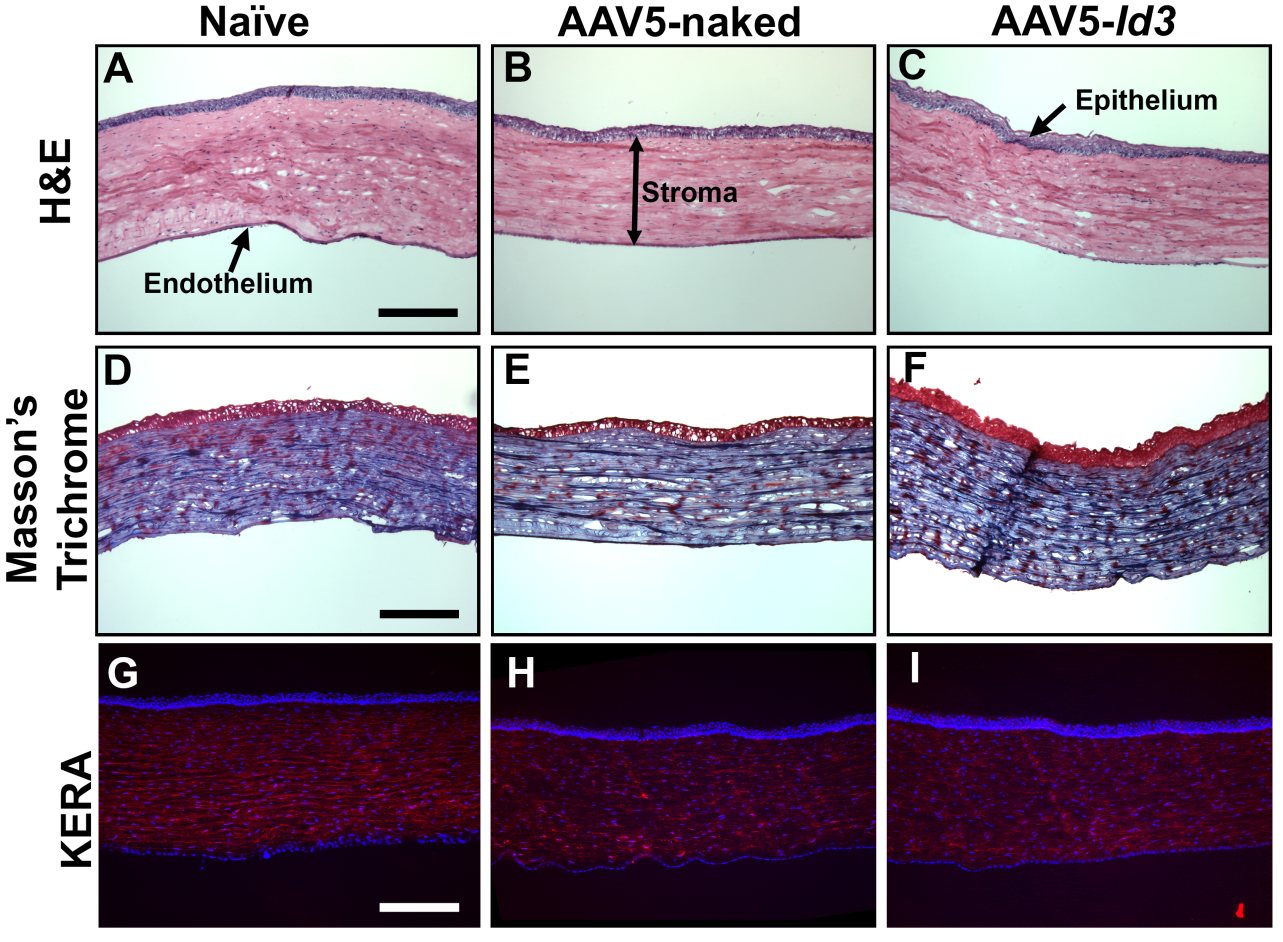
Histologic analysis of rabbit corneas at 7 months posttreatment showed no notable differences in collagen content, cellular architecture, or gross morphology across naive, AAV5-naked, and AAV5-*Id3* groups. H&E staining (**A–C**), Masson's trichrome staining (**D–F**), and Keratocan staining (**G–I**) demonstrated comparable corneal structure, supporting the safety and tolerability of AAV5-*Id3* gene therapy. Histologic findings at 2 months (data not shown) were similar. *Scale bar*: 100 µm (**A–I**).

The histopathologic examinations of H&E staining, Masson's trichrome staining, and DAPI immunofluorescence revealed that *Id3* gene therapy is tolerable and safe to the eyes (Fig. 8). The H&E images showed no visible morphologic changes or infiltration of inflammatory cells in the naive, AAV5-naked, and AAV5-*Id3* corneal tissue sections (Figs. 8A–C). Likewise, Masson's trichrome staining of naive, AAV5-naked, and AAV5-*Id3* corneal tissue sections revealed no appreciable differences in the gross collagen level (Figs. 8D–F). In addition, KERA staining, a cornea-specific keratan sulfate proteoglycan, along with DAPI, showed that kerotocyte density, cellular organization, and nuclear density in the corneas of the three groups looked similar (Figs. 8G–I).

### Effect of AAV5-*Id3* on Proinflammatory, Profibrotic, and Proangiogenic Markers

**Figure 9.**
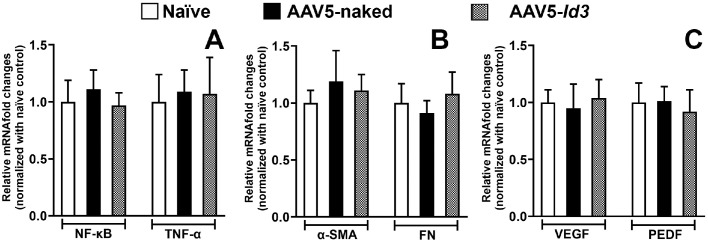
qRT-PCR analysis of proinflammatory (**A**), profibrotic (**B**), and proangiogenic (**C**) gene expression in naive, AAV5-naked, and AAV5-*Id3* rabbit corneas at 7 months posttreatment. No significant changes were observed in the expression of NF-κB, TNF-α (A), α-SMA, FN (B), or VEGF, PEDF (**C**) across groups. Statistical comparisons were performed using one-way analysis of variance with a Bonferroni post hoc test (*P* > 0.05).

The impact of AAV5-naked and AAV5*-Id3* on corneas was judged by measuring proinflammatory, profibrotic, and proangiogenic marker mRNA levels 7 months after euthanasia using qRT-PCR (Fig. 9). The mRNA levels of proinflammatory NF-κB, TNF-α (Fig. 9A), profibrotic α-SMA, FN (Fig. 9B), proangiogenic VEGF, and PEDF (Fig. 9C) were similar in the corneas of the naive, AAV5-naked, or AAV5*-Id3* groups. This suggested that AAV5*-Id3* gene therapy to the cornea is safe and tolerable.

### Delivered *Id3* Gene Copy Quantification

**Figure 10.**
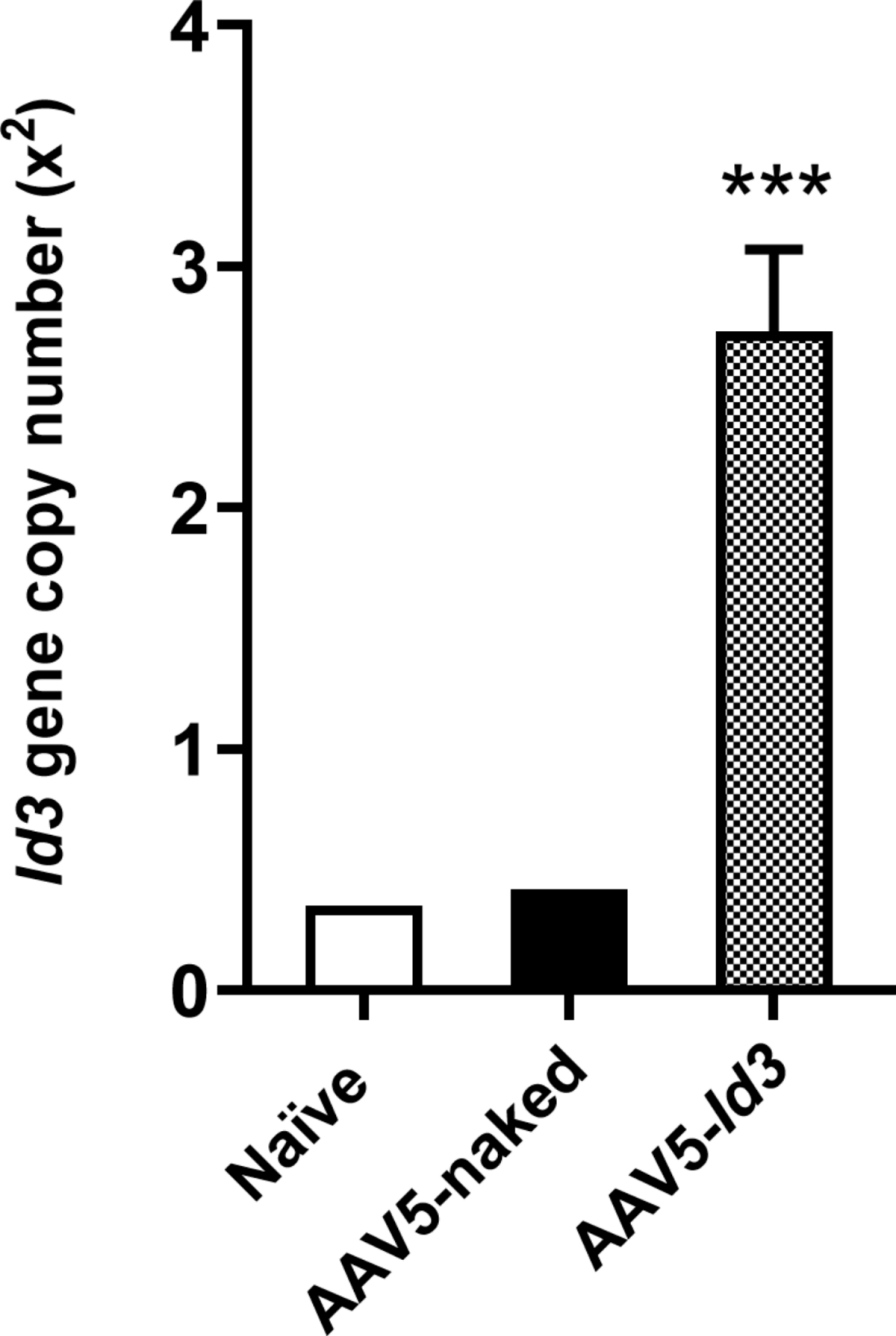
qRT-PCR analysis showing *Id3* gene copy number in naive, AAV5-naked, and AAV5-*Id3* rabbit corneas at 7 months posttreatment. AAV5-*Id3*–treated eyes showed a significantly higher *Id3* gene copy number compared to controls (***P < 0.001, one-way analysis of variance with Bonferroni post hoc test). *Error bars* represent ± standard error of the mean.

AAV5*-Id3*–treated rabbit corneas demonstrated 2.73 × 10^2^ ± 0.34 copies of the *Id3* gene per 1 µg DNA (Fig. 10). Conversely, the naive and AAV5-naked–treated corneas showed very low endogenous levels of *Id3* gene expression at 7 months.

## Discussion

Gene therapy has the potential to treat corneal diseases without needing frequent applications to the eyes. The cornea's avascular, transparent nature, combined with its immune privilege and ease of access for noninvasive evaluations, makes it ideal for gene therapy.^41^^–^^43^ In the past two decades, substantial progress has been made in showing the usefulness of gene therapy modalities to treat corneal conditions, including inherited dystrophies, allograft rejection, herpetic keratitis, corneal haze or fibrosis, and corneal neovascularization.^12^^–^^18^^,^^41^^–^^50^ In our customized gene delivery technique, we have carefully removed the corneal epithelium by gently scraping at 45° from the surface of the cornea in one direction, and Supplementary Figure S1 shows barely induced keratocyte apoptosis in the rabbit cornea in vivo. In this gene therapy method, as expected, the cornea with an intact epithelium showed no keratocyte apoptosis.^12^^–^^18^^,^^41^^–^^50^ Previous studies characterized the expression of Id proteins in corneal tissue and reported their role in regulating the differentiation of stromal fibroblasts using a gain-of-function approach.^28^^,^^29^ The findings suggest that *Id3* gene augmentation in the stromal fibroblasts prevents TGFβ-mediated conversion of corneal keratocytes to myofibroblasts through E-box protein. The AAV5-mediated *Id3* gene therapy, reducing injury-driven corneal fibrosis development in rabbits in vivo, was also reported.^13^ These studies highlight the promise of AAV5-*Id3* gene therapy for clinical translation if long-term safety and tolerability are proven.

The present study revealed the long-term safety, tolerability, and nontoxic nature of AAV5-*Id3* gene therapy to the eye in vivo using a rabbit model and a combination of multimodal clinical eye imaging, histology, immunofluorescence, and qRT-PCR results. The AAV5-*Id3* gene therapy was well tolerated and caused no apparent adverse effects to the cornea and other ocular surface tissues at 7 months. The biomicroscopy tools revealed no significant alterations in corneal architecture or morphological deviations in corneal epithelial, stromal, or endothelial cells (Figs. 1^2^,^3^,^4^,^5^–6). In addition, Supplementary Figure S2 is well corroborated by Figures 1 to 5 and shows no significant changes (*P* > 0.05) in the fluorescein uptake in corneal epithelium between the groups. Likewise, no changes in CCT, IOP, and tear volume were recorded over the course of the study (Fig. 7). The modified McDonald–Shadduck test depicted similar results (Table 2). The posteuthanasia histologic and molecular analyses of AAV5-*Id3* delivered corneas revealed tested parameters to naive or AAV-naked delivered corneas, further supporting the safety and tolerability of AAV5-*Id3* gene therapy (Figs. 8^9^–10).

The *Id3* gene may interact with the basic region of the TATA box E-box protein while playing a role in regulating differentiation events during trauma.^13^^,^^29^ However, it is reported that *Id3* gene augmentation under nontraumatic conditions does not disrupt normal cellular morphology, proliferation, or growth at the morphological or molecular levels.^13^^,^^29^ The Id proteins, including Id3, lack the basic region of the bHLH protein that prevents them from directly binding to DNA.^12^^,^^28^^,^^51^^,^^52^ This study did not examine a direct cause-and-effect relationship between *Id3* and E-box proteins. Further, the changes in many cellular parameters, such as proliferation, migration, differentiation, and growth factors or cytokines influencing corneal wound healing, have not been studied while evaluating the tolerability and safety of AAV*-Id3* gene therapy, which are potential limitations of this study. Additional studies are warranted to explore the full-scale toxicological effects of *Id3* gene augmentation in the cornea/eye.

Taken together, the findings of the present study suggest that a single topical AAV5-*Id3* gene treatment is tolerable, nontoxic, and safe to the cornea and ocular surface tissues. Previous studies demonstrating the success of AAV5-*Id3* gene augmentation in reducing corneal fibrosis in vivo suggest that topical tissue-targeted AAV5-*Id3* gene therapy may offer a promising therapeutic option for corneal scarring in vivo without significant short- or long-term toxicity to the eyes.

## Supplementary Material

Supplement 1
