# Supplementary material for: Long-Term Tolerability and Safety of AAV5-Id3 Gene Therapy to Eyes
Source: Transl Vis Sci Technol. 2026 Jan 28;15(1):38. doi: 10.1167/tvst.15.1.38 (PMC12859705; doi:10.1167/tvst.15.1.38)
Supplement: Supplement 1 [file tvst-15-1-38_s001.docx]

**
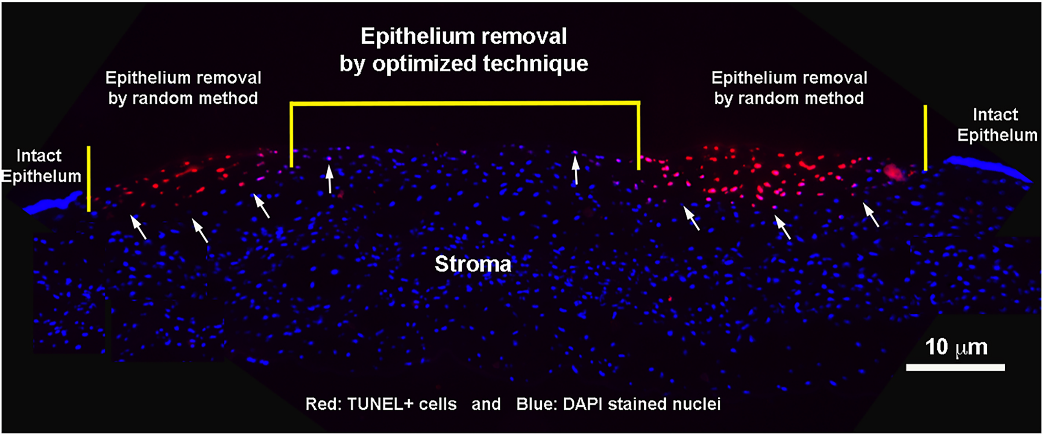
**

**Supplementary Figure S1.** A customized vector-delivery technique to facilitate gene delivery into rabbit stroma *in vivo* by passing epithelial and keratocyte apoptosis barriers. The image is taken at 100× magnification. Scale bar = 10 μm.

**
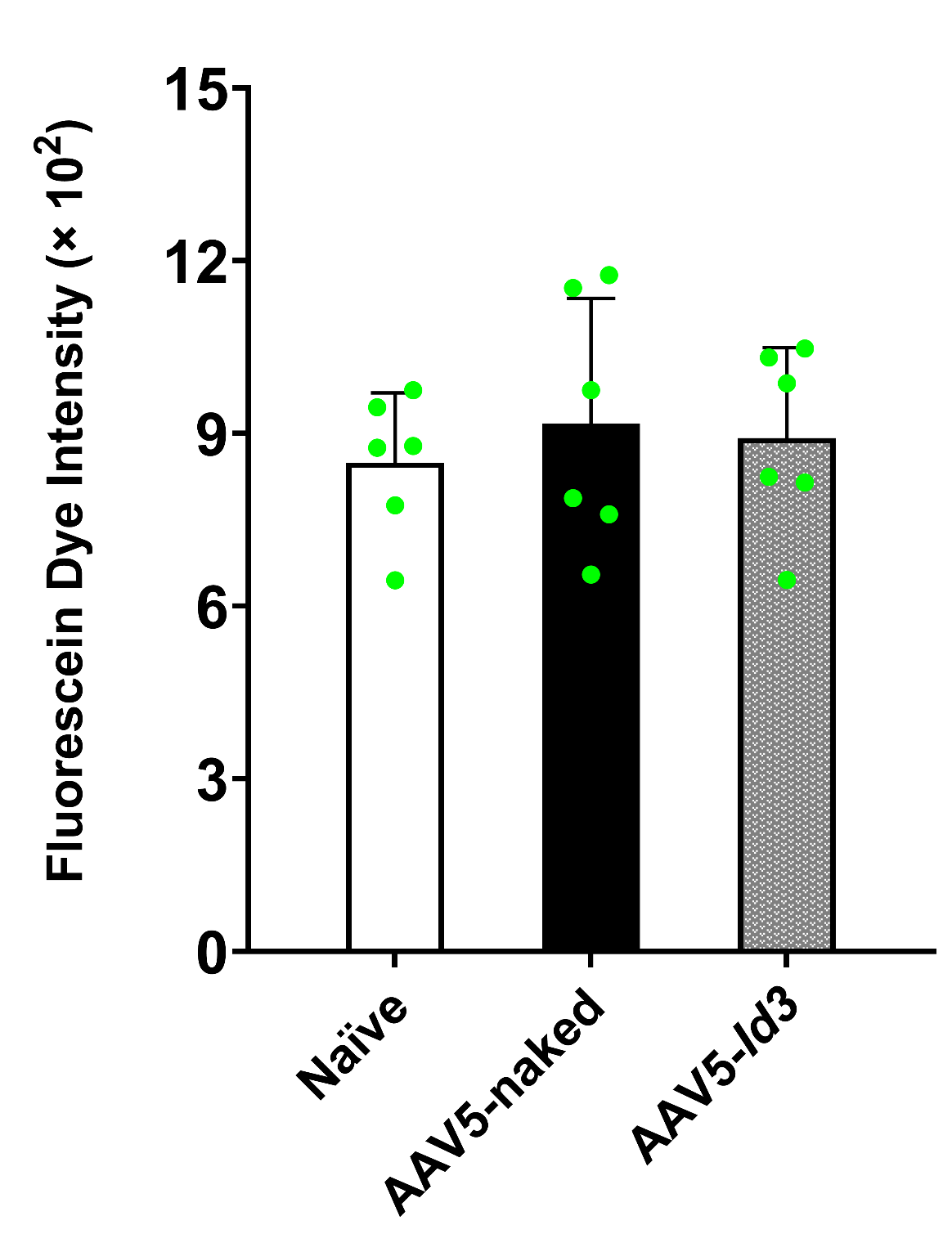
**

**Supplementary Figure S2.** Bar graphs showing fluorescein dye uptake intensity in the central corneal region in rabbit eyes of naïve, AAV5-naked, and AAV5-*Id3* rabbit eyes. No significant differences were observed among groups, demonstrating the corneal epithelium's health and showing the safety and tolerability of AAV5-*Id3* gene therapy *in vivo*. Statistical comparisons were performed using one-way ANOVA with Tukey’s post hoc test (P > 0.05).
